# Mechanically recyclable melt-spun fibers from lignin esters and iron oxide nanoparticles: towards circular lignin materials†

**DOI:** 10.1039/d3gc02381h

**Published:** 2023-09-14

**Authors:** Unnimaya Thalakkale Veettil, Adrian Moreno, Alberto J. Huertas-Alonso, Mohammad Morsali, Ievgen V. Pylypchuk, Li-Yang Liu, Mika H. Sipponen

**Affiliations:** a Department of Materials and Environmental Chemistry, Stockholm University Svante Arrhenius väg 16C SE-106 91 Stockholm Sweden mika.sipponen@mmk.su.se; b Wallenberg Wood Science Center, Department of Materials and Environmental Chemistry, Stockholm University SE-10691 Stockholm Sweden

## Abstract

The inferior thermoplastic properties have limited production of melt-spun fibers from lignin. Here we report on the controlled esterification of softwood kraft lignin (SKL) to enable scalable, solvent-free melt spinning of microfibers using a cotton candy machine. We found that it is crucial to control the esterification process as melt-spun fibers could be produced from lignin oleate and lignin stearate precursors with degrees of esterification (DE) ranging from 20–50%, but not outside this range. To fabricate a functional hybrid material, we incorporated magnetite nanoparticles (MNPs) into the lignin oleate fibers by melt blending and subsequent melt spinning. Thermogravimetric analysis and X-ray diffraction studies revealed that increasing the weight fraction of MNPs led to improved thermal stability of the fibers. Finally, we demonstrated adsorption of organic dyes, magnetic recovery, and recycling *via* melt spinning of the regular and magnetic fibers with 95% and 83% retention of the respective adsorption capacities over three adsorption cycles. The mechanical recyclability of the microfibers represents a new paradigm in lignin-based circular materials.

## Abstract 2

Melt spinning of lignin C18 fatty acid esters in the presence of magnetite nanoparticles provides a facile and green way to magnetically responsive microfibers for water purification and more.

## Graphical abstract

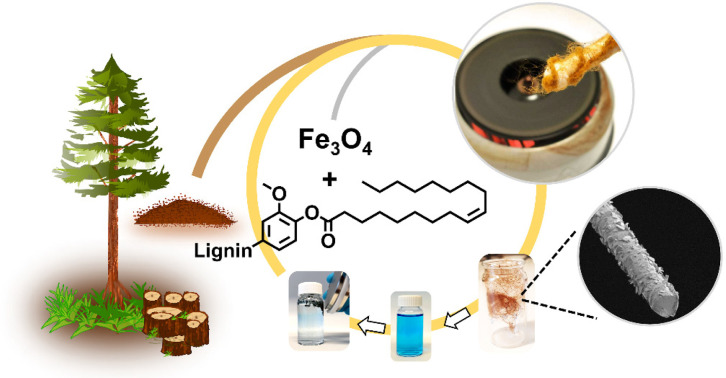

## Introduction

Lignin, as a natural polyphenol, imparts structural rigidity and hydrophobicity to secondary cell walls of terrestrial plants,^1^ and is a major by-product of biorefineries and pulping industries.^2–4^ Driven by expanding electrochemical and lightweight composite materials markets,^5,6^ melt spinning^7,8^ and electrospinning^9^ of carbon fiber precursors from kraft lignin has caught interest. In the kraft pulping process, wood chips are cooked at elevated temperatures (160–170 °C) to solubilize lignin in the form of phenolic oligomers and polymers with a number average molecular weight below 10 kDa and a glass transition temperature (*T*_g_) at 150–180 °C.^10,11^ However, inferior thermoplastic properties and particularly the lack of distinct melting point renders kraft lignin poorly suited for melt spinning.

Esterification can decrease the glass transition temperature and improve thermoplastic behaviour of lignin.^12–14^ This process converts the free hydroxyl groups to esters with tunable hydrocarbon chains, increases the free volume of lignin macromolecules, as well as improves the thermal mobility of the product.^15^ Short chain esters such as lignin butyrate, lignin methacrylate and lignin propionate have been employed for the preparation of unsaturated thermosets with improved solubility in styrene^16^ towards high-performance engineering materials, ranging from thermoplastics to carbon fibers.^17^ Among long-chain esters, lignin laurate (C_12_) and lignin palmitate (C_16_) have been studied for the fabrication of composites^12^ and coating formulations for fiber-based packaging.^18^ Softwood kraft lignin esterified with unsaturated tall oil fatty acids has also been covalently attached onto cellulose fibers to fabricate antimicrobial cellulosic fibers.^19^ Softwood kraft lignin was chemically modified using bio-sourced oil *via* reactive extrusion, improving its thermoplastic properties. This modified lignin made it applicable for melt-blending with poly(butylene adipate-*co*-terephthalate).^20^ Similarly, lignin esters were found to be usable as lubricants and plasticizers,^21^ as well as in colored polyesters for containers exhibiting lower UV light transmittance,^22^ packaging^18^ and components of polystyrene blends.^23^

Formulation with lignin-based plasticizers^24^ and using solvent-fractionated lignin^25^ have been used to achieve spinnability of lignin, but the literature is lacking examples on fiber production using lignin esters. In view of large-scale production, triglyceride oils are among the most plausible sources of C_12_–C_20_ fatty acids. Selecting the appropriate acyl chain length for lignin esters is challenging due to the unpredictability of their spinnability based solely on their physicochemical properties. It would be beneficial to have the option of experimentally testing the melt spinning of modified lignin grades in the laboratory to accelerate the decision-making process. This is especially significant because large-scale spinning equipment is often unavailable, which results in a lack of literature on the materials chemistry and processability of lignin esters in fiber production. These limitations have impeded the development of scalable processes for producing lignin-based fibers.

In the present work, we used an affordable cotton candy machine for melt-spinning of esterified lignin. The facile screening of the fiber production allowed us to discover an optimal range of degree of esterification that produces lignin microfibers in gram scales within a few minutes. We also demonstrate incorporation of magnetite nanoparticles (MNPs) into lignin oleate fibers (LOFs) *via* the melt-spinning process, which is advantageous compared to previously reported chemical synthesis methods.^26,27^ Moreover, using anionic, cationic, and uncharged dyes as model pollutants, we also present the hybrid fibers as magnetically recoverable and mechanically recyclable adsorbents for wastewater treatment. We note that many lignin esters have been synthesized prior to this work, especially for blending with synthetic polymers. However, the majority of earlier efforts mainly aimed for extensive esterification with high degrees of esterification, varying properties by changing the acyl chain length. In contrast, our approach controls the thermoplastic properties of softwood kraft lignin by achieving a moderate degree of esterification between 20 and 50%, which reduces the levels of residual fatty acid byproducts.

## Results and discussion

**Fig. 1:**
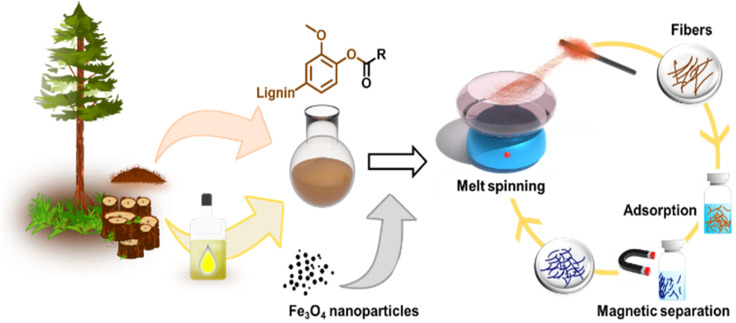
General approach to synthesize thermoplastic lignin esters and their blends with magnetite nanoparticles for production of melt-spun fibers, their use as adsorbents, magnetic recovery and mechanical recycling. SKL was esterified in an anhydrous organic solvent using oleoyl chloride or stearoyl chloride.

The primary objectives of this study were twofold: firstly, to assess the suitability of softwood kraft lignin (SKL) esters for melt spinning and evaluate how the degree of esterification (DE) affects this process, and secondly, to explore the possibility of enhancing the fibers by incorporating inorganic materials that provide added functionalities. To accomplish these goals, our approach for producing melt-spun lignin esters and their nanocomposites with magnetite nanoparticles is illustrated in Fig. 1.

### Fabrication of lignin ester microfibers *via* melt-spinning

**Fig. 2:**
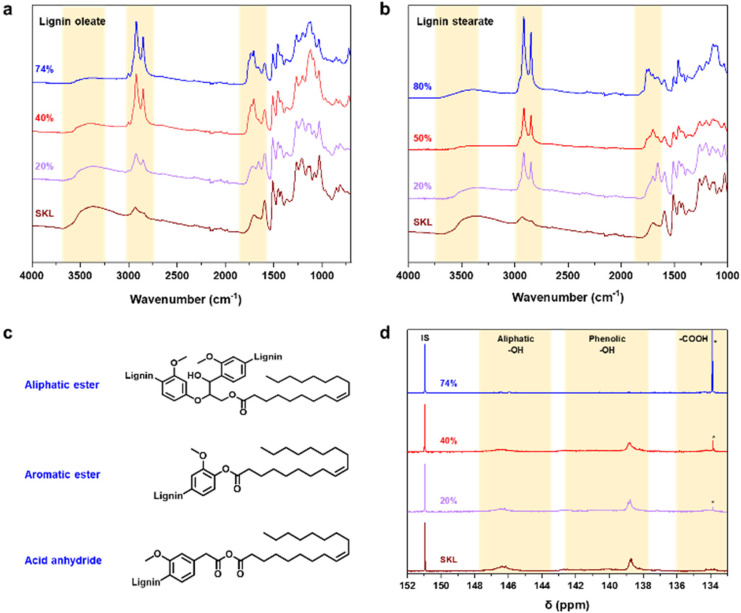
Characterization of (a) lignin oleate, and (b) lignin stearate by FTIR spectroscopy. (c) Possible structures, and (d) ^31^P NMR spectra of lignin oleate. IS denotes internal standard and * free oleic acid.

To render lignin thermoplastic for melt spinning, we first modified the SKL *via* base-catalyzed esterification using oleoyl chloride and stearoyl chloride.^14,28^ We tested different degrees of esterification to find a product with a melting point and glass transition temperature that allows melt spinning. The synthesis of esterified softwood kraft lignin was monitored using ATR-FTIR spectroscopy shown in Fig. 2a and b. The decrease in intensity of the bands at 3400 cm^−1^ (O–H stretching) corroborated that the hydroxyl groups of lignin reacted successfully. Moreover, the increased intensities of the bands at 2850–2950 cm^−1^ (C–H stretching) and 1700 cm^−1^ (C=O stretching) indicated the incorporation of the aliphatic chain and ester functional groups derived from the fatty acids, respectively.^14,28^ The hydroxyl content (phenolic and aliphatic) of SKL and esterified lignin were determined by quantitative ^31^P NMR spectroscopy shown in Fig. 2d. The total hydroxyl content of SKL was found to be 5.85 mmol g^−1^, which is essentially similar to our previously published result from the same batch of SKL, of 5.94 mmol g^−1^ ^28^ and slightly lower compared to 6.67 mmol g^−1^ previously reported by Koivu *et al.*^14^ for SKL.

The amount of hydroxyl groups decreased gradually according to the increasing DE, which again confirmed the successful esterification process. Details of the hydroxyl contents of the esterified lignin are summarized in Table S1 in the ESI.†^31^P NMR analysis revealed that the amount of unreacted oleic acid present in the sample is the highest in the case of 74% DE of lignin oleate, which is found to be 16% (Table S1†). For 20 and 40% DE of lignin oleate the calculated amount of unreacted oleic acid is only 2%. The molecular weight of the SKL and esterified lignin were investigated using GPC shown in Fig. S1.† The molecular weight distribution curves of the lignin esters showed a shift towards the higher molecular weight region compared to that of the SKL, thus confirming the increment on molecular weight as a consequence of the esterification process.

**Fig. 3:**
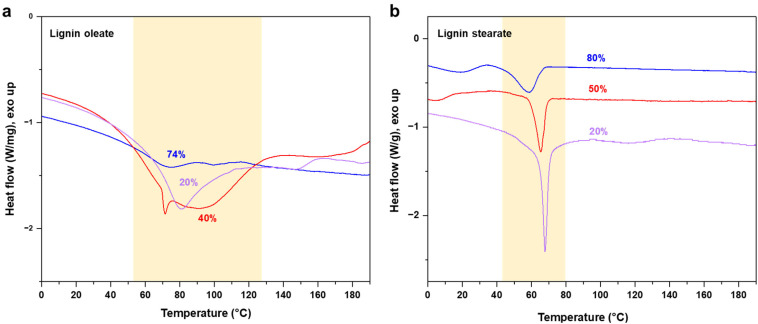
DSC thermograms of esterified SKL with different degrees of esterification: (a) lignin oleate, and (b) lignin stearate.

The target of the esterification process was to improve the thermal properties of SKL, as it typically possesses a poorly distinguishable glass transition temperature (*T*_g_) in the range of 90 to 180 °C and no clear melting point as shown in Fig. S2.† In contrast to the pristine SKL, the lignin esters exhibited a melting point in the range of 60 to 90 °C shown in Fig. 3a and b. Interestingly, the melting peak of lignin oleate was sharpest with the lowest degree of esterification (20%), with broadening at 40% and finally being poorly discernible at 74%. The peak broadening observed for lignin oleate was probably because of the double bond present in the alkyl chain which allow certain degree of rotation, while in the case of lignin stearate the rigid alkyl chain restricts rotation within the alkyl chain. As a result, the melting peaks occurred at lower temperatures compared to comparable DE of the lignin oleates shown in Fig. 3b.

**Fig. 4:**
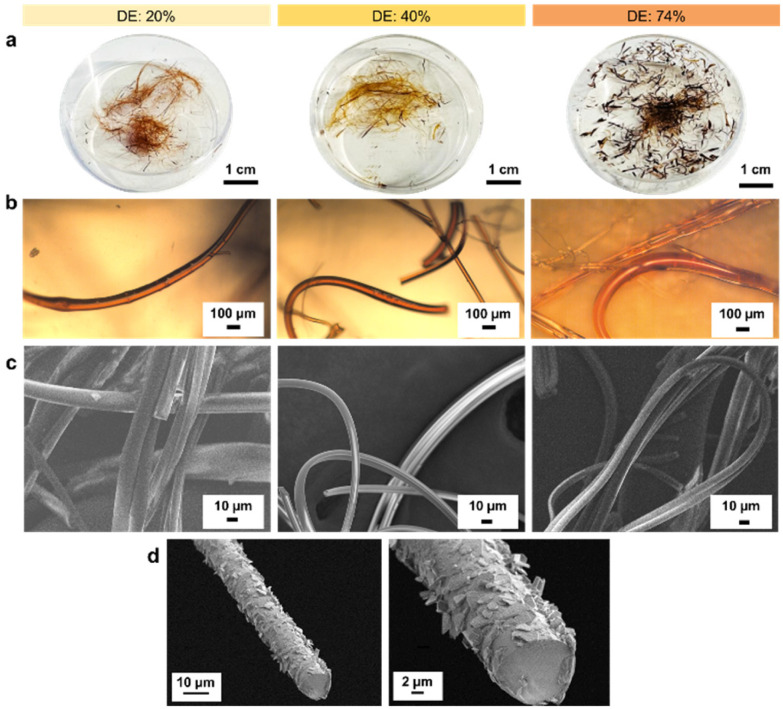
(a) Digital images, (b) optical microscopy images and (c) SEM images of microfibers formed by melt spinning lignin oleate having a degree of esterification (DE) of 20%, 40% and 74%. (d) SEM images of cross-section of LOFs with DE 40% at different magnifications.

With increasing DE, the melting point of the lignin oleate fibers (LOFs) decreased and there were clear differences in the appearances of the fibers shown in Fig. 4a. With the lowest DE (20%), the fibers appeared brown and short, with 40% DE the fibers became more yellowish and longer, and lastly the fibers corresponding to 74% DE became more blackish brown and were flaky due to their low melting point as noted above in the thermal characterization Fig. 3a. The flaky appearance of the fibers at the highest DE was also evident from optical microscopy investigation shown in Fig. 4b and made them prone to and re-melt on the collector walls. The average diameters of the LOFs were found to be 14, 20 and 8 μm for the lignin esters with DE of 20, 40 and 74%, respectively. When studied in scanning electron microscope (SEM) the fibers revealed a rich array of surface and cross-sectional morphologies shown in Fig. 4c and d. In addition to lignin oleate, the microfibers could be successfully prepared from lignin stearate precursors as well, see Fig. S3 and S4.† Lignin oleate fibers at DE of 40% were used for further examination due to their aforementioned properties. The cross-section of freeze-fractured LOFs with DE 40% revealed the presence of surface decoration by irregularly shaped platelets with sizes from about 1 μm to 5 μm shown in Fig. 4d. Though beyond the scope of this work, we speculate that these platelets arise from the fraction of lignin with the highest melting point or hydrophobicity, and therefore deposit on the fiber surfaces.

LOFs with 40% DE were tested for stability in water, acetone, ethanol, and 1 M ammonium hydroxide after 1 hour and 24 hours at room temperature as shown in Fig. S5.† Water-immersed fibers were stable, while fibers in acetone dissolved rapidly and completely. Fibers in ethanol partially dissolved after one hour but not completely after 24 hours. Restricting the degree of esterification to 40% preserved 3.53 mmol g^−1^ total hydroxyl groups, making lignin increasingly hydrophobic. Ethanol and 1 M ammonium hydroxide only partially dissolved the LOFs within 24 hours. The solubility of lignin in aqueous alkali increases with increasing pH in the range 7–13. The fibers were stable from pH 1 to pH 7 even after 24 hours. At pH 9, there was a swelling point where the fibers were partially dissolved and sedimented. At pH 13, the fibers partially dissolved due to saponification of the fatty acid esters and deprotonation of the phenolic hydroxyl groups. These results are in accordance with the literature,^29^ where the authors studied the lignin dissolution in several aqueous alkaline solvents. In general, the solubility of biopolymers in organic solvents often increases upon replacing the hydroxyl groups with ester groups, but it is not yet clear that to what extent the solubility changes upon increasing the alkyl chain length.^30^

### Multifunctional fibers *via* incorporation of magnetite nanoparticles

Due to the observed stability of the fibers within the pH range 1–7 shown in Fig. S5,† we were interested in using them as adsorbents for wastewater treatment. For this application, it is essential to effectively separate the adsorbents from the treated water. One of the most attractive strategies is to use magnetically assisted separation processes.^31–34^ In fact, the incorporation of MNPs to polymers has gained extensive attention due to their physicochemical and magnetic properties as well as the ease of combination with organic or inorganic compounds.^35,36^

**Fig. 5:**
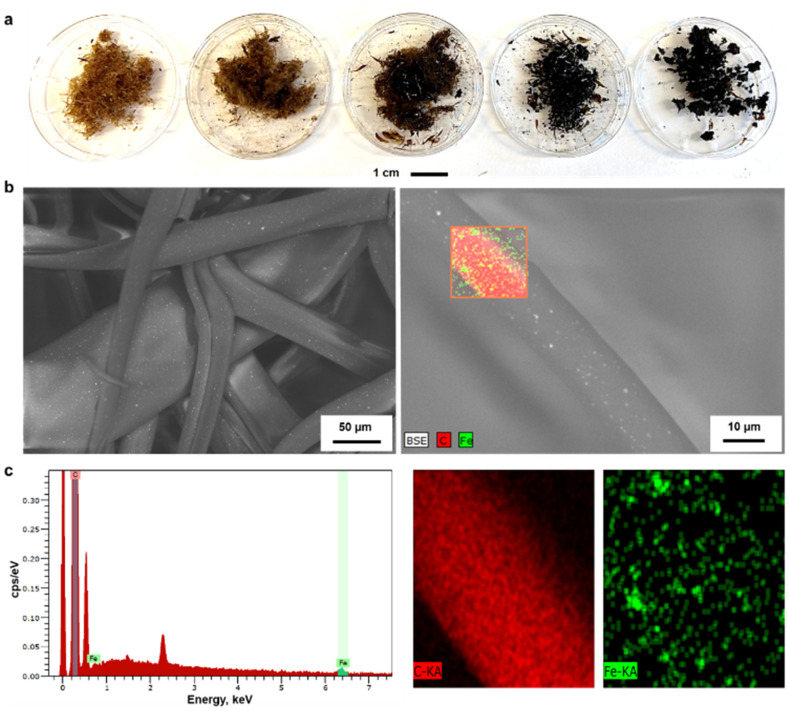
(a) Digital images of magnetite incorporated lignin oleate having 40% DE with magnetite weight percentage of 1.0, 2.0, 4.8, 9.1 and 16.6% from left to right respectively, (b) SEM Images and (c) EDS analysis of magnetite incorporated lignin oleate fibers with 4.8 wt% magnetite nanoparticles.

With this inspiration, we prepared hybrid lignin-magnetite fibers to facilitate the recovery of the used adsorbents by magnetic means. The incorporation of the MNPs was done by melting lignin oleate (LO, 40% DE) at 80 °C in the presence of 1.0, 2.0, 4.8, 9.1 and 16.6 wt% of MNPs with respect to the weight of the entire mixture. Then, the blend of LO-magnetite nanoparticles was used for melt-spinning. A general observation from this screening experiment was that the suitability to fiber formation after magnetite incorporation decreased when increasing the percentage of MNPs. Fibers formed with up to 4.8 wt% MNPs were longer than those formed by incorporating 9.1 and 16.6 wt% of MNPs shown in Fig. 5a.

The morphology of the LOFs that contained 4.8 wt% of MNPs was observed through SEM imaging. The average diameter of the hybrid fibers was found to be 40 μm by image analysis which is twice in diameter than the LOFs made from LO at 40% DE. The organic and inorganic components were uniformly distributed in the fibers according to the recorded SEM images that show the MNPs as bright spots on the fibers shown in Fig. 5b. Energy Dispersive X-ray Spectroscopy (EDS) was used for the elemental distribution analysis and mapping as shown in Fig. 5c and Table S2.† The incorporation of MNPs was confirmed by the peaks of Fe at 0.7 keV and 6.4 keV (Lα and Kα lines respectively), while the peaks of carbon were assigned at 0.27 keV (Kα line).

**Fig. 6:**
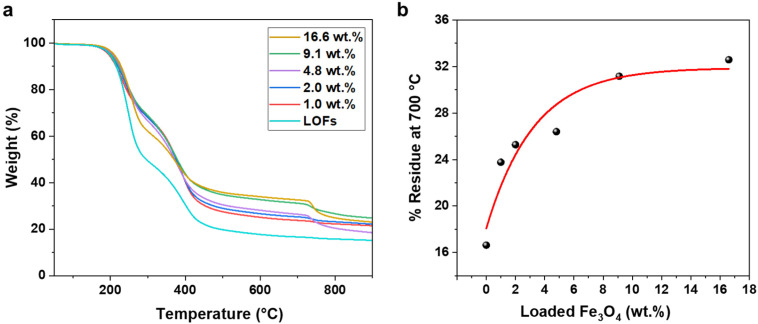
(a) Thermogravimetric analysis of lignin oleate fibers (LOFs) and the magnetic fibers with (MLOFs) loaded with various percentages of MNPs. (b) Weight percentage of residue at 700 °C as function of theoretical magnetite nanoparticle content of MLOFs.

The effect of MNPs on the thermal stability of the LOFs was further studied by thermogravimetric analysis (TGA). The hybrid fibers exhibited higher thermal stability compared to the LOFs as shown in Fig. 6a. Moreover, it was observed that the thermal stability of the hybrid lignin-magnetite fibers was dependent on the amount of magnetite incorporated to the hybrid fibers. Thus, the LOFs with the highest content of MNPs (16.6 wt%) exhibited the highest thermal stability, while the hybrid lignin fibers with the lowest magnetite content (1.0 wt%) exhibited the lowest thermal stability. The residue left at 700 °C was plotted against wt% of loaded MNPs and the experimental data was fitted exponentially in Fig. 6b. From the exponential fit it is clear that the incorporation of MNPs is possible up to a certain amount and that only after the level of 9.1 wt% incorporation of MNPs there is a significant trade-off in the efficiency, which leads to wastage of MNPs. To achieve a maximum magnetization and efficient incorporation of MNPs, we chose to focus on the lignin oleate fibers with 4.8 wt% MNPs incorporated.

**Fig. 7:**
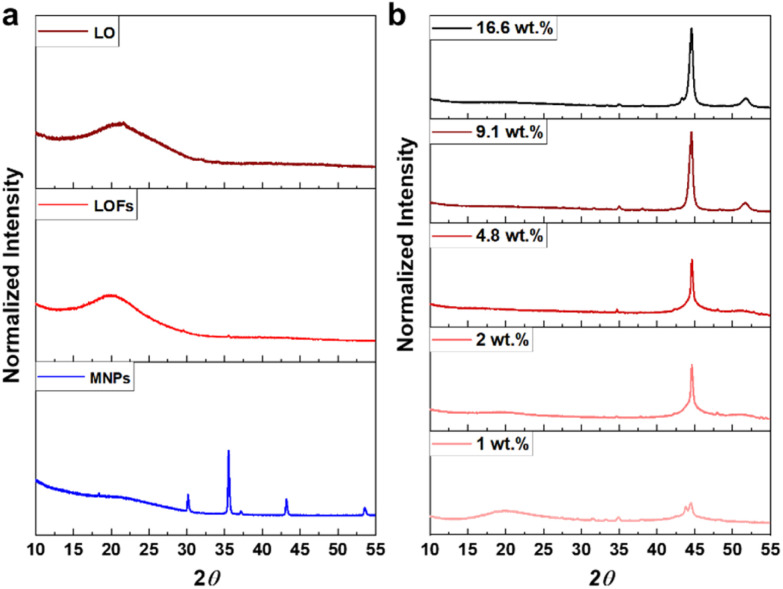
(a) XRD spectra of lignin oleate (LO), lignin oleate fibers (LOFs) and magnetite nanoparticles (MNPs) respectively and (b) XRD spectra of hybrid fibers with different wt% of magnetite nanoparticles.

The X-ray diffractograms of LO and LOFs showed a broad diffraction peak with a maximum at 2*θ* = 20°, corresponding to the amorphous structure of lignin shown in Fig. 7a. The characteristic peaks of MNPs were observed; (220) at 2*θ* corresponds to 30°, (311) at 35°, (400) at 42°, (422) at 53° and (511) at 57°, which is essentially concordant with the results from literature.^37^ Similar peaks were observed in LOFs that contained MNPs with the intensity of the peaks proportionally increasing until 4.8 wt% magnetite content shown in Fig. 7b and thereafter levelling off due to the saturation concentration, as was also observed with TGA as shown in Fig. 6a.

**Fig. 8:**
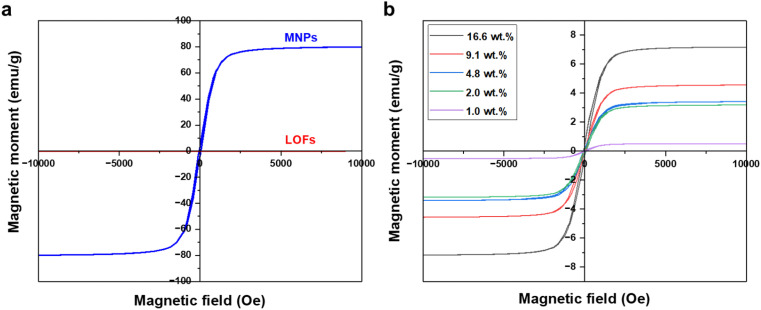
VSM Curves of (a) lignin oleate fibers (LOFs) and magnetite nanoparticles (MNPs) and (b) hybrid fibers with different wt% of magnetite nanoparticles.

The magnetic behavior of the fibers was measured using a vibrating sample magnetometer. Significant improvement in magnetization was observed for the MLOFs with LOFs. Compared to the saturation magnetization of 77 emu g^−1^ of the magnetite nanoparticles the magnetic moment for LOFs was close to zero, denoting the diamagnetic behavior of the sample (Fig. 8a). Meanwhile, the saturation magnetization (Ms) for the LOFs with 4.8 wt% MNPs content, which used for further studies was found to be 3.3 emu g^−1^. The magnetic moments were found to be increasing with increasing magnetite content from 1 wt% to 16.6 wt% as shown in Fig. 8b. However, the saturation magnetization levels of MLOFs were not proportional to the saturation magnetization of pure magnetite, but lower because of the diamagnetic behavior of the lignin ester. The fact that the major composition of the hybrid material is lignin oleate results in an increase of the diamagnetic thickness of the hybrid samples and hence the magnetic moment is shielded. The superparamagnetic behavior of the MNPs originates from their small size.^26^ In this study, Scherrer's equation^38^ was used to derive the average crystallite size of 25 nm, which is comparable to the particle size reported elsewhere.^39^

It is crucial to consider the principles of green chemistry when synthesizing materials that incorporate magnetite nanoparticles. Admittedly, such comparisons are subject to multidimensional discussions about the performance, magnetization, and engineering aspects of the production. Nevertheless, when comparing the present work to the prior published reports it becomes evident that the omission of organic synthesis and activations steps avoids the use of hazardous chemicals and simplifies the process, making it more feasible for potential scale-up (Table 1). On the other hand, higher contents of MNPs (up to 83%) have been achieved through chemical routes. However, as we demonstrate in the next section, fibers with 4.8 wt% magnetite content facilitate their magnetic separation in wastewater treatment, and enable mechanical recycling, which has not been reported with other lignin-magnetite materials to date.

**Table 1:** Comparison of different methods for Fe_3_O_4_ nanoparticles incorporation to lignin

Ref.	Chemicals	Hazard pictograms	*T* (°C) & reaction time	Prerequisite	Magnetite content	Magnetization
26	Dioxane : water (9 : 1, v/v)	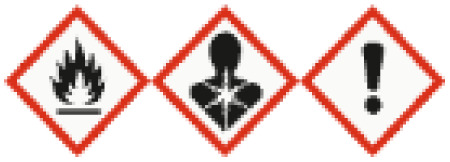	RT for 2 h	Activation of lignin by oxidation, performed in dark	16.6 wt%–83.3 wt%	∼5–30 emu g^−1^
	Sodium periodate	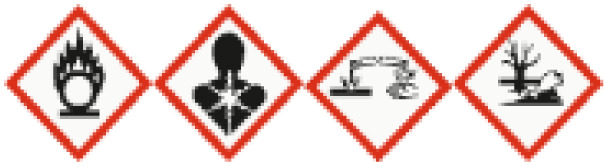				
27	Ethanol : water (4 : 1, v/v)	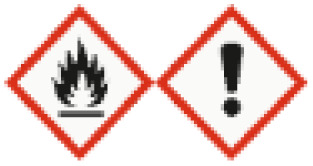	45 °C for 16 h; 60 °C for 6 h	Ar atmosphere and pH = 9 for the reaction	25.0 wt%–66.7 wt%	∼16–23 emu g^−1^
	Tetraethyl orthosilicate	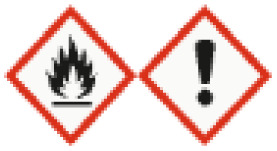				
	Formaldehyde	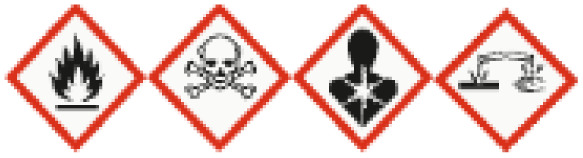				
	3-Aminopropyltriethoxysilane	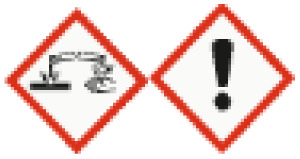				
	Aq. Ammonia	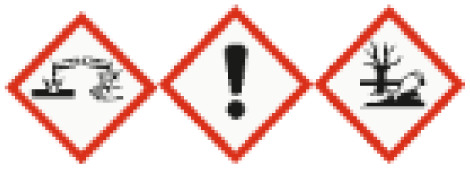				
40	Poly(ethylene)imine	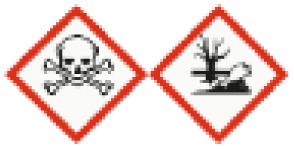	40 °C for 2 h for amino functionalization	Amino functionalization and stabilization of Fe_3_O_4_ nanoparticles	44.4 wt%	—
	Na-alginate emulsifier	—				
	3-Aminopropyltriethoxysilane	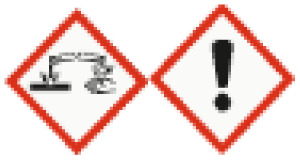				
	Oleic acid	—				
41	2-(*N*-Morpholino) ethanesulfonic acid	—	RT for 2 h	Preparation of PSA/SiO_2_/Fe_3_O_4_/AuNP microspheres (magnetic microspheres; MMS) and amination of lignin	—	—
	*N*-Hydroxysuccinimide	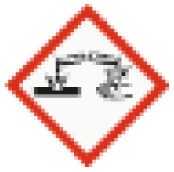				
	Triethylamine	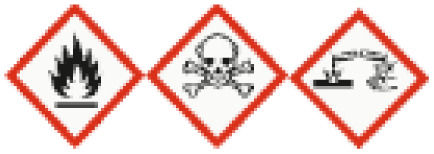				
	NaCl buffer	—				
	Methanol	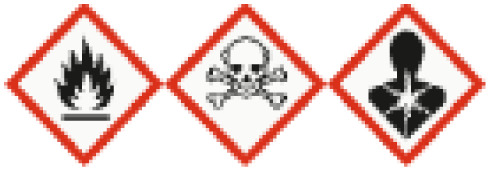				
	*N*-(3-Dimethylaminopropyl)-*N*′-ethyl carbodiimide	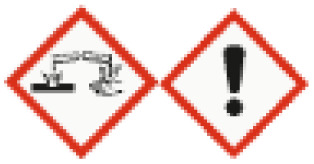				
This work	—	—	85 °C for 15–30 min	Melting of esterified lignin	1.0 wt%–16.6 wt%	∼0.5–7.1 emu g^−1^

### Towards recyclable adsorbents for wastewater treatment

Dyes from industrial processes, such as those from the textile industry, pollute aquatic systems and are posing environmental and ecological challenges to our society. In addition to the dyes, their metabolites are potential carcinogens and may cause allergic reactions in humans.^42,43^ Lignin microfibers were therefore evaluated as low-cost adsorbent for cationic, non-ionic and anionic dyes. The recyclability of the fibers as well as their magnetic recoverability was also investigated. The spent fibers were collected, dried and subjected to melt-spinning without desorption and used again as adsorbents with fresh dye solutions.

**Fig. 9:**
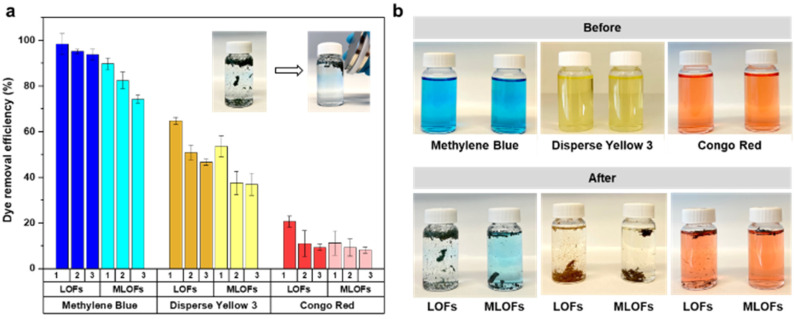
Lignin oleate fibers as recyclable adsorbents. (a) Dye removal efficiency of methylene blue, disperse yellow 3 and congo red by LOFs and MLOFs in three adsorption-recycling cycles. Inset: digital images showing magnetic recoverability of the fibers after methylene blue adsorption (b) digital images of dye solutions before and after adsorption.

LOFs had the highest adsorption affinity towards cationic methylene blue followed by disperse yellow 3 and lowest adsorption affinity towards anionic congo red (Fig. 9a). For methylene blue, LOFs had a removal efficiency of 98.4% in the first cycle and 95.2% and 93.8% in the second and third cycles, respectively. MLOFs exhibited lower removal efficiencies of 89.8% in the first cycle and 82.4% and 74.3% in the second and third cycles, respectively. This general trend was repeated in the case of the non-ionic dye disperse yellow 3, with LOFs showing a removal efficiency of 64.7% in the first cycle, which decreased to 50.8% and 46.6% in the second and third cycles, respectively. MLOFs had a removal efficiency of 53.5% in the first cycle, which decreased to 37.5% and 36.8% in the second and third cycles, respectively. In the case of the anionic dye congo red, LOFs had a removal efficiency of 20.5% in the first cycle, which decreased to 11.0% and 9.4% in the second and third cycles, respectively. MLOFs had a removal efficiency of 11.2% in the first cycle, which decreased to 9.3% and 8.2% in the second and third cycles, respectively.

From the above results it is evident that LOFs are more efficient adsorbents compared to the corresponding magnetite incorporated fibers, irrespective of the type of dye as shown in Fig. 9a. A possible reason for this difference is the observation that LOFs tend to break into small fibrils, which increased their surface area in the dye solutions, whereas the corresponding hybrid fibers with MNPs remained more rigid and did not disintegrate to a same extent. The magnetic separation of the fibers with the help of a magnet (150 mT) allowed recovering even smaller spent fibers that could be effectively used as precursor for recycled melt-spun fibers, shown in the Videos S1–S3 in ESI.† As there is growing interest in circular lignin-based materials,^44^ LOFs and MLOFs seem to be promising candidates for such materials.

LOFs and MLOFs were also evaluated for their heavy metal removal efficiency and potential to facilitate oil/water separation. These fibers displayed modest equilibrium adsorption capacities. Specifically, the equilibrium adsorption capacity for Cu^2+^ ions from a 10 g L^−1^ copper(ii) sulphate solution was measured at 1.0 mg g^−1^ for LOFs and 1.3 mg g^−1^ for MLOFs following a 24-hour experiment (refer to Fig. S6 in the ESI†). The low adsorption capacities might stem from hydrophobic surfaces that lack adsorption sites, which hinders in-depth exploration of heavy metal adsorption. In contrast, we have showcased how a filter mat composed of lignin oleate microfibers enables effective oil–water separation. By filtering an olive oil and water mixture through the fiber mat, we achieved a 96% water recovery, while the oil phase was retained by the filter (Fig. S6†).

## Conclusions

We have established a facile method for the fabrication of lignin-based microfibers *via* laboratory melt-spinning that allowed rapid screening of structure–property relationships. To render softwood kraft lignin (SKL) thermoplastic and render its thermal properties according to the needs of the melt spinning, we made a successful synthesis of lignin stearate and lignin oleate as proven by spectroscopic and thermal analyses. The esterification significantly improved thermal processability of SKL *via* melt spinning. We discovered that degrees of esterification between 20–50% ensure thermoplastic behavior to produce fibers with appropriate length and diameter, whilst extensive esterification rendered esterified lignin unsuitable for melt-spinning. Here it is also important to mention that esterification of lignin with fatty acids is already implemented in industry,^45^ while also greener esterification routes have been reported,^46,47^ encouraging the potential scalability of these fibers for future applications. At 40% degree of esterification, magnetite nanoparticles were successfully incorporated into the melt spun fibers, which improved their thermal stability and allowed using them as magnetically recoverable adsorbents. In addition, we demonstrated easy regeneration and reusability of the fibers as a proof of concept of circular lignin materials and could lead to other applications beyond adsorbents such as recyclable composites. Achieving these partial esterification levels in industrial setups like reactive extrusion reactors could yield advantages, such as low levels of unreacted reagents and fatty acid byproducts. Although confirming this hypothesis is beyond the scope of our current study, we hold the view that solvent-free esterification without work-up procedures would significantly promote green chemistry in this domain. Further work is also needed to scale up the fiber production and improve their mechanical properties towards structural applications.

## Experimental section

### Materials

The softwood kraft lignin (SKL) was obtained from UPM (BioPiva™100, Finland), and dried at 60 °C for 12 hours before use. Tetrahydrofuran (THF), N,N′-Dimethylformamide (DMF) and dichloromethane (DCM) were purchased from Honeywell. Stearoyl chloride (>97%) was purchased from Tokyo Chemical Industry (TCI). Anhydrous pyridine was purchased from Carlo Erba. Methylene blue was purchased from Thermo Fisher. The oleoyl chloride, sodium sulphate, iron oxide (Fe_3_O_4_) nanoparticles, disperse yellow 3 (dye content 30%), congo red dyes, and anhydrous copper(ii) sulphate were purchased from Sigma Aldrich. Olive oil was purchased from Garant. THF, DMF, methyl 5-(dimethylamino)-2-methyl-5-oxopentanoate and pyridine were stored under inert conditions, with molecular sieves and purged with nitrogen gas before performing the experiments. All the other chemicals were used as received.

### Synthesis of oleoyl and stearyl esterified lignin

The procedure for base-catalyzed esterification of SKL was adapted from Koivu *et al.*^14,28^ Briefly, 5.0 g of SKL (5.85 mmol g^−1^ of total phenolic and aliphatic hydroxyl groups, measured by quantitative ^31^P NMR analysis), was dissolved in 15 mL of THF and 7.5 mL of DMF at 45 °C under stirring. We note that the esterification reaction can be successfully carried out in methyl 5-(dimethylamino)-2-methyl-5-oxopentanoate (Rhodiasolv® PolarClean) as a greener alternative to DMF, while keeping the reaction conditions similar.^48^ When lignin was dissolved, 3.8 mL of pyridine (9.4 mmol g^−1^ of lignin) was added to the mixture at 45 °C under nitrogen, and left to react for 30 minutes. The amount of acyl chloride (oleoyl or stearyl) added was varied according to the targeted degree of esterification (DE). For 20% degree of esterification (% DE) 1.26 mmol of acyl chloride, for 40% DE 2.53 mmol of acyl chloride, for 52% DE 3.16 mmol acyl chloride, for 74% DE 5.06 mmol of acyl chloride and for 80% DE 8.22 mmol acyl chloride per one gram of lignin was injected using a syringe and the reaction was kept for 24 hours at 45 °C under nitrogen with sufficient stirring. The DEs were determined using ^31^P NMR Spectroscopy. After completion of the reaction, the reaction mixture was transferred to a separation funnel with 40 mL of DI water, and the esterified lignin was extracted with dichloromethane (3 × 80 mL). Then, the organic phases were combined, dried over sodium sulphate and finally, dichloromethane was evaporated under reduced pressure at 45 °C. The products were found in the form of solid powder or viscous fluid depending as function of the degree of esterification. In general, higher degree of esterification affords viscous black lignin-fatty acid esters, while lower degree of esterification affords black powder lignin-fatty acid esters.

### Preparation of magnetite incorporated esterified lignin

Physical melt blending approach was used for preparing hybrid samples. By heating lignin oleate (LO) powder at DE of 40% above 85 °C, the Fe_3_O_4_ nanoparticles were blended with the lignin oleate in 1.0%, 2.0%, 4.8%, 9.1% and 16.6%, relative to the weight of the entire mixture. The mixture was blended well and cooled down to solidify the blended mixture.

### Fabrication of lignin-based melt-spun microfibers

The melt-spun microfibers were produced using a cotton candy machine (Champion Nordics, CHSVM110). It consists of an annular heating tube and a heating plate. The cotton candy machine was preheated to approximately 200 °C for three minutes prior to the use. Then, by turning off the machine and thereby the spinning, esterified lignin was added to the heating plate and spinning was continued after turning on the cotton candy machine. The microfibers formed were collected using the back side of the cotton candy spoon. Magnetite incorporated lignin oleate fibers (MLOFs) were formed the same way by adding the blended mixture to the heating plate and collected the same way.

### Batch dye adsorption experiment

The dye adsorption was carried out using three different dyes; cationic (methylene blue), non-ionic (disperse yellow 3) and anionic (congo red) dye. The dye solutions were made in deionized water with a concentration of 5 mg L^−1^ to study the equilibrium adsorption capacity of the fibers. The batch adsorption experiments were performed using 50 mg of the synthesized LOFs and MLOFs (based on lignin oleate at 40% DE) in vials containing 20 mL of dye solutions. Equilibrium concentrations of the dye solutions after dye adsorption were measured after 24 h agitation at room temperature at a rate of 60 rpm (Incu-ShakerTM, Fisherbrand). After adsorption, the LOFs were separated *via* centrifugation (1000 rpm for 10 min using Universal 320 centrifuge) by excluding the supernatant and MLOFs were separated using a magnet (150 mT). The spent fibers were dried and used for second cycle of dye adsorption by regenerating the fibers using melt-spinning without desorbing the dye. The experiment was continued until third cycle of dye adsorption and each experiment was replicated twice. Absorbance measurements of dye solutions were performed using a UV–vis spectrophotometer (Genesys 150 UV-Vis spectrophotometer, Thermoscientific) with cuvettes (path length l = 1 cm) at time 0 and after 24 h, conducted at wavelength *λ* = 664 nm (methylene blue), *λ* = 357 nm (disperse yellow 3) and *λ* = 496 nm (congo red). The equilibrium adsorption capacity (*Q*_e,adsorption_) of the fibers was measured using the following equation:1
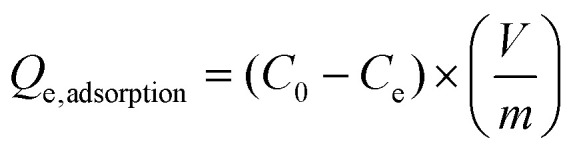
dye removal efficiency was calculated using the following equation:2
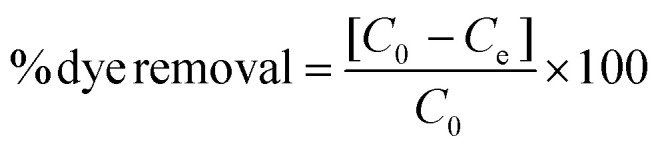
*C*_0_ is the concentration of the initial dye solution, *C*_e_ is the equilibrium concentration of the dye, *V* is the volume of the dye solution, and *m* is the mass of the adsorbent in g.

### Heavy metal adsorption

The heavy metal removal experiment was carried out using copper(ii) sulphate solution. The adsorption experiments were performed using 20 mg of the synthesized LOFs and MLOFs in vials containing 15 mL of 10 g L^−1^ CuSO_4_ solution. The copper sulphate concentration was determined initially and after 24 h based on its absorbance at *λ* = 814 nm using a UV–vis spectrophotometer (Genesys 150 UV-Vis spectrophotometer, Thermoscientific). The adsorption experiments were conducted at room temperature using shaking incubator (Incu-ShakerTM, Fisherbrand), with the same method described for batch dye adsorption experiment above and eqn (1).

### Oil/water separation

A fiber mat is made into the form of filter using lignin oleate fibers and used for separation of olive oil and water. The mixture of olive oil and water was prepared in a 20 mL vial by 1 : 11 w/w ratio and filtered through the lignin microfiber filter. After filtration, the water phase was recovered and calculated the % recovery of water with respect to amount of water in the original mixture.

### ATR-FTIR spectroscopy

Attenuated Total Reflectance Fourier Transform Infrared (ATR-FTIR) spectroscopy was used for the analysis of functional groups in the SKL and esterified lignin samples, by using a Varian 610-IR Spectrometer equipped with a diamond ATR Optics. The spectra were measured from 400 to 4000 cm^−1^ with a total of 32 scans.

### Quantitative ^31^P NMR spectroscopy

^31^P NMR spectra were recorded using a 500 MHz spectrometer (Bruker BioSpin GmbH) operating at 500.13 MHz for ^1^H nucleus and at 202.47 MHz for ^31^P nucleus. Acquisition parameters were: pulse angle 90°, inverse-gated proton decoupled pulse sequence for suppressing NOE, relaxation delay 5 s, number of scans 128, acquisition time 1 s and spectral width of 80 645 MHz. Samples were prepared by dissolving 30 mg of lignin in a solvent mixture of 0.1 mL of CDCl_3_ and 0.3 mL of pyridine, followed by addition of 0.15 mL of DMF, 0.2 mL of *endo-N*-hydroxy-5-norbornene-2,3-dicarboximide as internal standard, and 0.05 mL of chromium(III) acetylacetonate as relaxation agent. Then, 2-chloro-4,4,5,5-tetramethyl-1,2,3-dioxaphospholane (TMDP, 0.15 mL) was added was phosphitylation reagent. The fully soluble reaction mixture was stirred at room temperature for 10 minutes and transferred to an NMR tube. The spectra were recorded within 1 hour from the sample preparation.

### Differential scanning calorimetry

Differential Scanning Calorimetry (DSC) was used to determine the thermal properties of SKL and esterified lignin with different degree of esterification, using a Netzsch DSC 214 Polyma instrument. The program used a heating and cooling rate of 10 °C min^−1^, the purge gas was N_2_. The program included a ramp from 20 °C to −50 °C, isothermal at −50 °C for 10 min, ramp from −50 °C to 200 °C, isothermal at 200 °C for 25 min, and finally cooling to 20 °C.

### Thermogravimetric analysis

Thermogravimetric analysis (TGA) was used to study the thermal degradation behavior of esterified lignin and magnetite incorporated esterified lignin using a Discovery TGA with a platinum HT pan, using 10–20 mg of samples. The temperature ramp was from 10 °C to 900 °C with a heating rate of 10 °C min^−1^ under inert atmosphere.

### Gel permeation chromatography

Molecular weight analysis was performed by Gel Permeation Chromatography (GPC, SECcurity2, PSS), consisting of a pump, a column oven set at 60 °C, and a variable wavelength detector (VWD). 10 mg of lignin samples were dissolved in 1 mL DMSO/LiBr (0.5 wt%, HPLC Grade, Fisher Scientific), following with the filtration using a PTFE membrane filters (0.22 μm, Whatman Uniflo). For analysis, a sample volume of 50 μL was injected manually to the system and fractionated by a series of PSS GRAM columns (one guard column, two 1000 Å columns, and one 30 Å column) with eluting solvent DMSO/LiBr at a flow rate of 1 mL min^−1^. Molecular weight analysis was performed using a WinGPC software based on a series of polystyrene sulfonate standards from 1.1 to 976 kDa.

### Electron microscopy

The morphology of esterified lignin microfibers was investigated using an optical microscope (Nikon, Alphaphot2) with a 10X and a 50X focus lenses. Additionally, a JEOL 7000F Scanning Electron Microscope (SEM) operated at 10 kV was used to study fibers sputtered by a thin Au layer for 60 s before examination to reduce the charging effect. The morphology of magnetite incorporated esterified lignin fibers was investigated using HITACHI-TM3000 SEM operated at an accelerating voltage of 15 kV. Energy Dispersive X-ray spectroscopic (EDS) mapping was taken on the SEM with Bruker Quantax 70 EDX Spectrometer.

### X-ray diffraction

Powder X-ray diffraction (XRD) patterns of the samples were obtained using a D8 Discover Diffractometer in reflection mode using Cu Kα radiation (*λ* = 1.5418 Å), 2*θ* ranging from 5° to 65° with an increment of 0.01 and rotation speed of 15 rotations per min. The average crystallite size was found using Debye–Scherrer equation:3
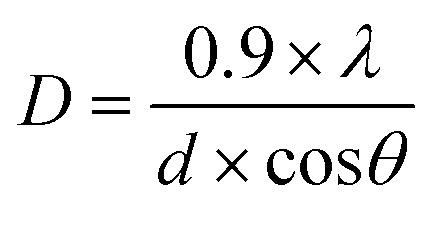
where *λ* is the wave length (nm), *D* is the crystallite size (nm), *d* is the full width at half maximum intensity of the peak (radians) and *θ* is the Bragg's diffraction angle

### Magnetization measurements

The magnetic properties of the samples were measured on a physical property measurement system (PPMS) from Quantum Design (USA). The vibrating Sample Magnetometer, 40 Hz (VSM) option was used for temperature and field dependence (DC), performed in high vacuum from −1 to 1 T. Samples were mounted on polypropylene pucks that were inserted into the measuring chamber using a brass sample holder and connected at the bottom of the Dewar to the measuring systems.

## Author contributions

U. T. V.: investigation, formal analysis, visualization, writing – original draft; A. M.: conceptualization, methodology, investigation, writing – review & editing; A. J. H. A.: validation, writing – review & editing; I. V. P.: formal analysis, writing – review & editing; M. M.: visualization, writing – review & editing; L. L.: validation, writing – review & editing; M. H. S.: conceptualization, funding acquisition, methodology, project administration, supervision, writing – review & editing.

## Conflicts of interest

The authors declare that they have no conflict of interest.

## Supplementary Material

GC-025-D3GC02381H-s001

GC-025-D3GC02381H-s002

GC-025-D3GC02381H-s003

GC-025-D3GC02381H-s004
