# Supplementary material for: Mechanically recyclable melt-spun fibers from lignin esters and iron oxide nanoparticles: towards circular lignin materials
Source: Green Chem. 2023 Sep 14;25(24):10424–35. doi: 10.1039/d3gc02381h (PMC10711735; doi:10.1039/d3gc02381h)
Supplement: GC-025-D3GC02381H-s001 [file GC-025-D3GC02381H-s001.pdf]

Electronic Supplementary Material (ESI) for Green Chemistry.
This journal is © The Royal Society of Chemistry 2023

Supporting Information

Mechanically recyclable melt-spun fibers from lignin esters and iron oxide nanoparticles: towards circular lignin materials

Unnimaya Thalakkale Veettil,[a] Adrian Moreno,[a, †] Alberto J. Huertas-Alonso,[a] Mohammad Morsali, [a, b] Ievgen V. Pylypchuk,[a] Liyang Liu,[a] Mika H. Sipponen[a, b, *]

[a]: Department of Materials and Environmental Chemistry, Stockholm University, Svante Arrhenius väg 16C, SE-106 91 Stockholm, Sweden

[b]: Wallenberg Wood Science Center, Department of Materials and Environmental Chemistry, Stockholm University, SE-10691 Stockholm, Sweden

[†] Present address: Laboratory of Sustainable Polymers, Department of Analytical Chemistry and Organic Chemistry, University Rovira I Virgili, Tarragona 43007, Spain

**Corresponding author:* *mika.sipponen@mmk.su.se*

This PDF contains:

Supplementary Tables S1-S2

Supplementary Figures S1-S6

Table S1. ^{31}P-NMR spectroscopic analysis of SKL and its oleate esters.

Sample	Aliphatic -OH (mmol/g)	Aromatic -OH (mmol/g)	Total -OH (mmol/g)	Unreacted oleic acid/sample mass (%)
SKL	1.86	3.99	5.85	-
SKL, 20% DE	1.14	3.52	4.66	2
SKL, 40% DE	0.93	2.60	3.53	2
SKL, 74% DE	0.46	1.06	1.52	16

SKL, softwood kraft lignin, DE, degree of esterification.

Table S2. Elemental map of relative intensities based on SEM-EDS analysis of MLOFs.

El	AN	Series	Net unn. C	norm. C	Atom. C	Error
C	6	K-series	82.02	82.02	86.35	8.8
O	8	K-series	16.82	16.82	13.29	2.2
S	16	K-series	0.59	0.59	0.23	0.0
Fe	26	K-series	0.57	0.57	0.13	0.0
Total			100	100	100	

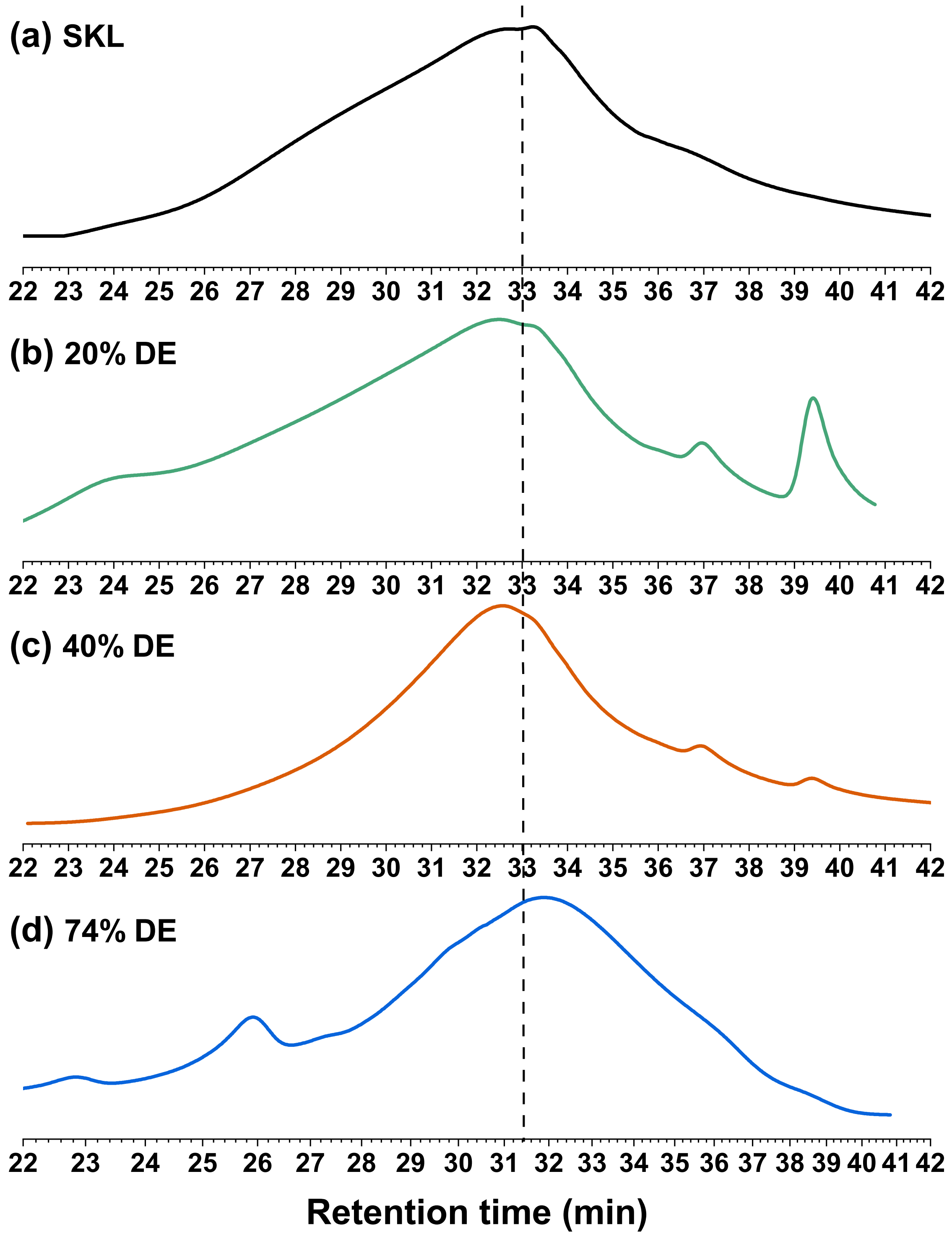

Figure S1. Gel permeation chromatogram of lignin oleates with degrees of esterification (a) 0% - original SKL; (b) 20%; (c) 40%; and (d) 74%.

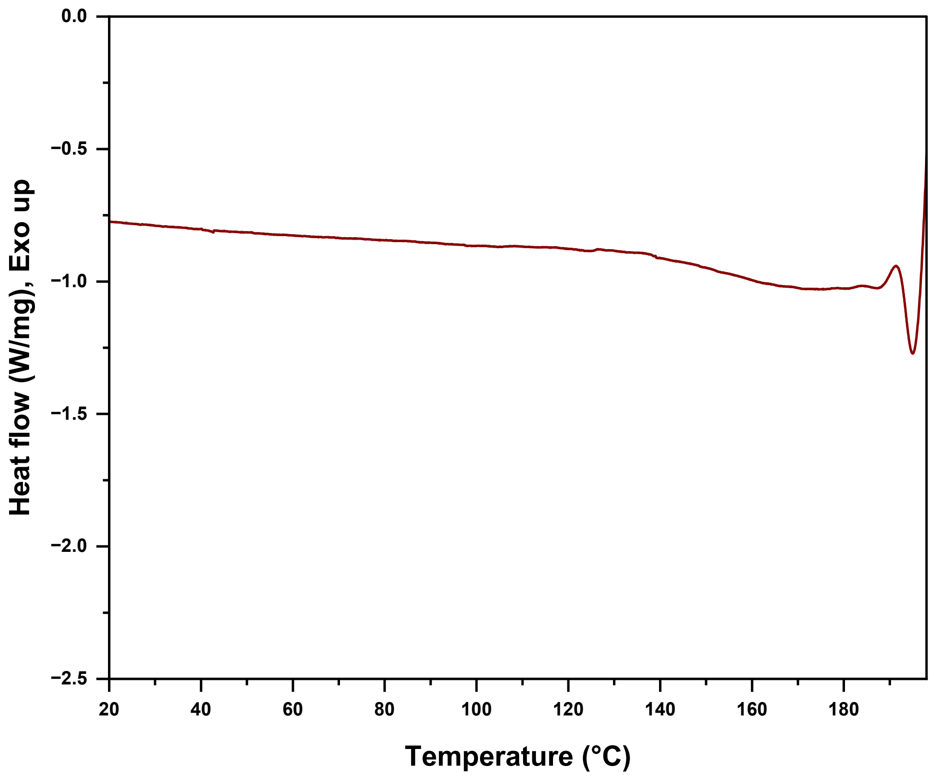

Figure S2. DSC thermogram of softwood kraft lignin.

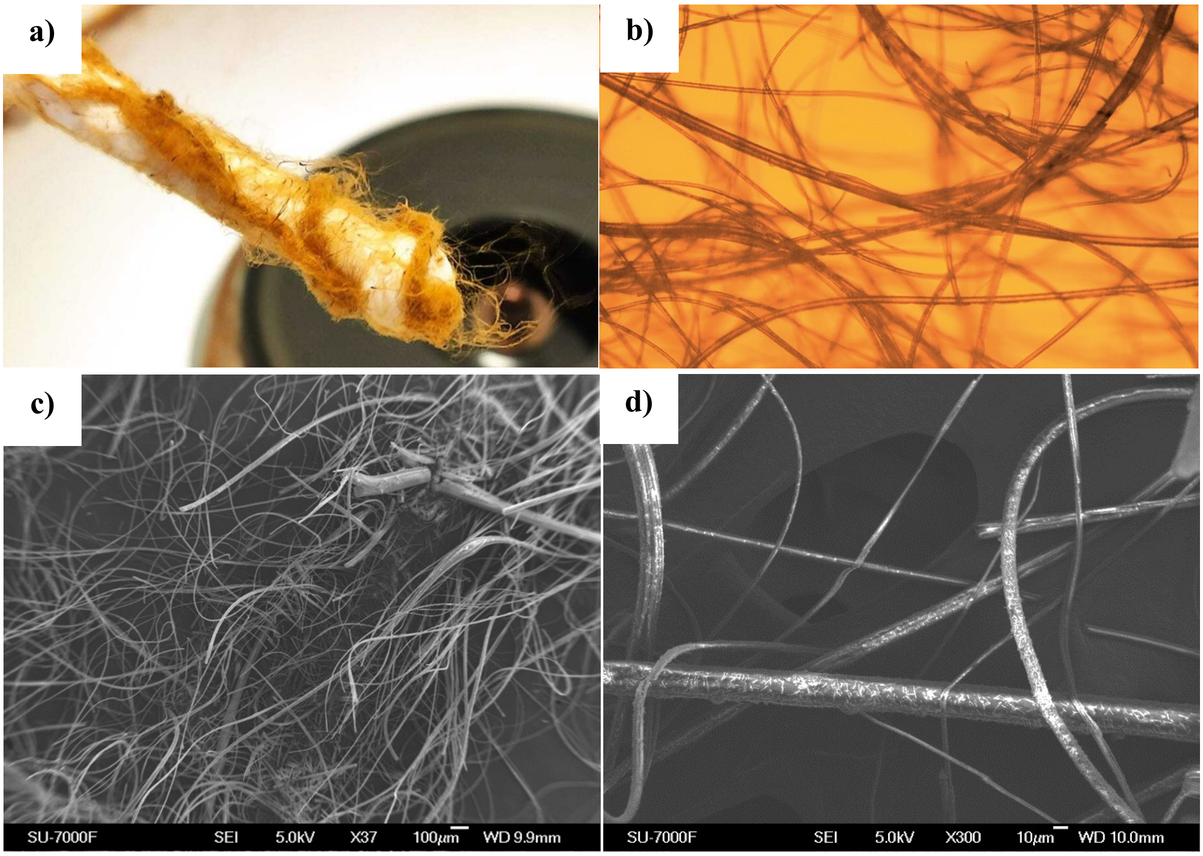

Figure S3. Lignin oleate fibres with 50% DE: a) A digital image; b) An optical microscopy image; c) and d) SEM images.

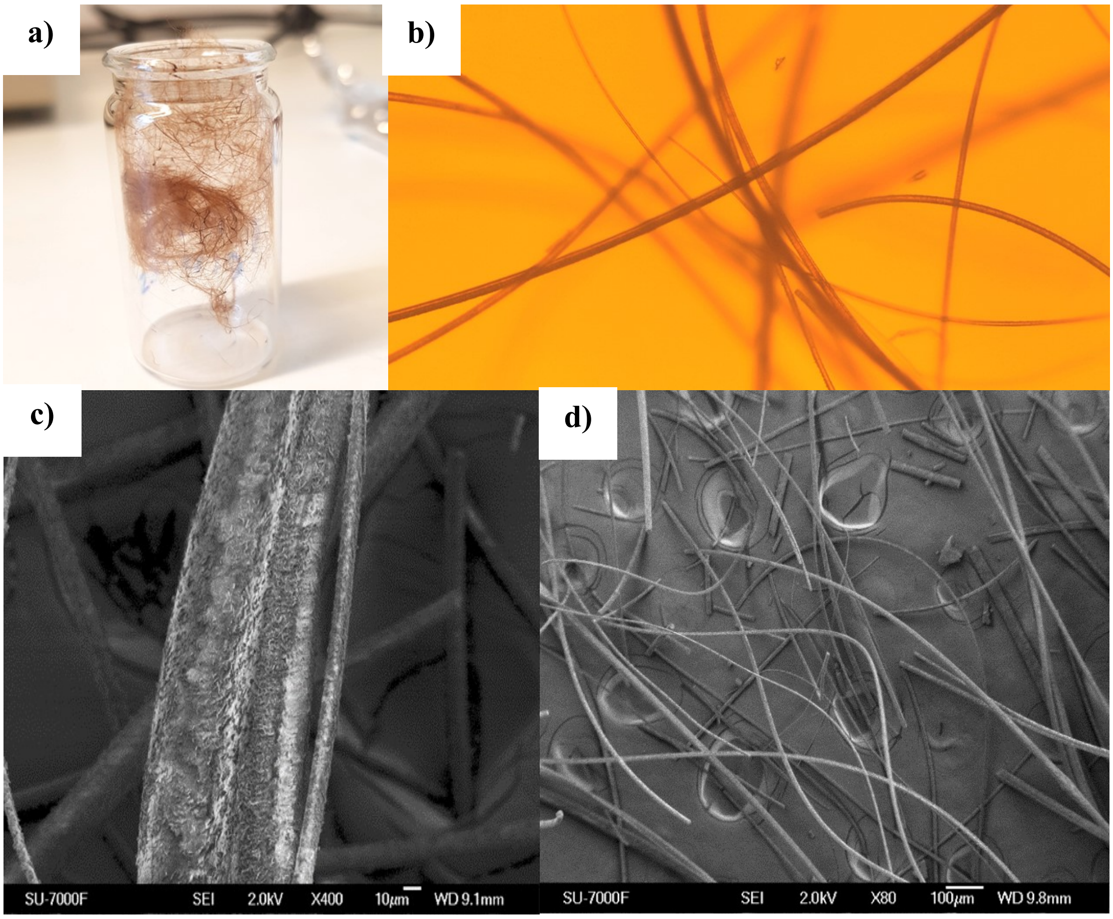

Figure S4. Appearance of fibers prepared from lignin stearate at 20% DE (a) A digital photograph; (b) An optical microscopy image, b) c) and d) are SEM images of fibres using 20% DE using stearoyl chloride.

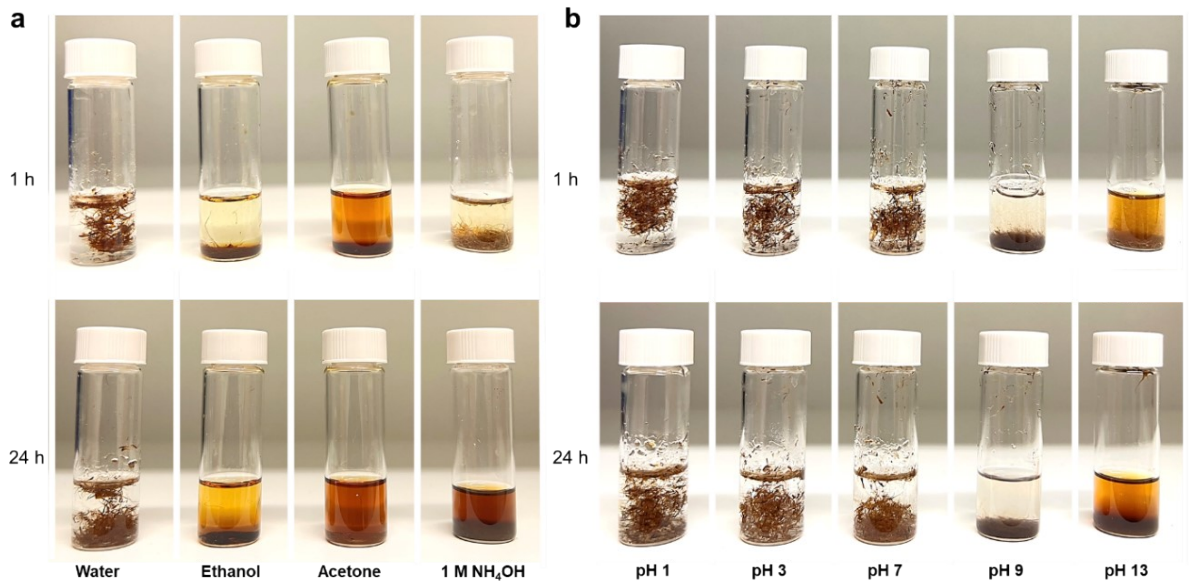

Figure S5. Stability of lignin oleate microfibers (DE= 40 %) as followed by their visual appearance in (a) different solvents and (b) aqueous media at different pH. The height of the vial is 5.5 cm.

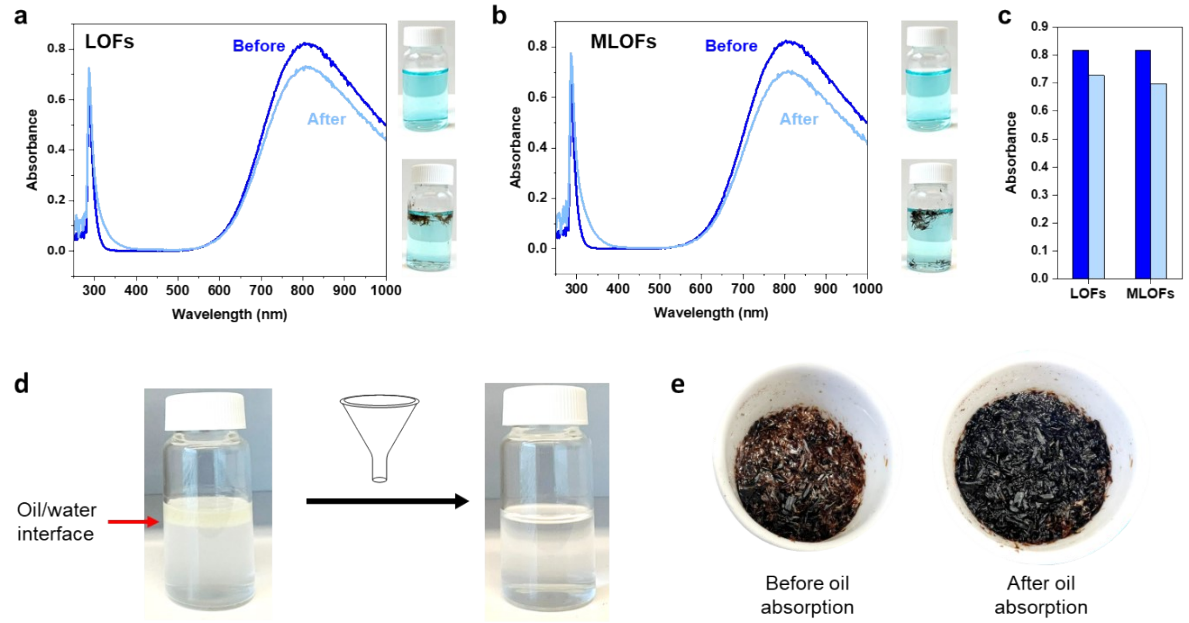

Figure S6. UV-Vis spectra of Cu^{2+} adsorption by (a) lignin oleate microfibers (DE= 40%), (b) magnetite incorporated lignin oleate fibers, (C) Bar chart showing comparison of UV-Vis absorbance of LOFs and MLOFs at before (dark blue) and after (light blue) 24 hours of adsorption and (d) Separation of olive oil from water using LOFs as filter. (e) Appearance of the lignin microfiber filter map before and after oil/water separation.
